# The callus fracture sign: a radiological predictor of progression to hypertrophic non-union in diaphyseal tibial fractures

**DOI:** 10.1007/s11751-015-0238-y

**Published:** 2015-11-24

**Authors:** S. Salih, C. Blakey, D. Chan, J. C. McGregor-Riley, S. L. Royston, S. Gowlett, D. Moore, M. G. Dennison

**Affiliations:** Department of Trauma and Orthopaedics, Northern General Hospital, Herres Rd, Sheffield, S5 7AU UK; Department of Radiology, Northern General Hospital, Sheffield, UK

**Keywords:** Ilizarov technique, Tibial fracture, Fracture healing, Radiography, X-ray, Hypertrophic non-union

## Abstract

We report a radiological sign which predicts progression to hypertrophic non-union for fractures of the tibial diaphysis. Radiographs of 46 tibial fractures were reviewed independently by four orthopaedic trauma surgeons and two musculoskeletal radiologists. Patients were identified from a database of tibial fractures managed with Ilizarov frame fixation. There were 23 fractures that progressed to non-union requiring further surgery. The controls were 23 fractures that had united without need for further surgery at 1-year follow-up. Radiographs selected were the first images taken following frame removal. All radiographs were anonymised and randomized prior to review. Presence of the callus fracture sign was identified in 16 radiographs of the fractures that progressed to non-union, and 7 of the united fracture group. Sensitivity is 69.6 %. Specificity is 91.4 %. Positive and negative predictive values are 88.9 and 75.0 %, respectively. These results compare favourably with computerised tomography for predicting non-union. Intra- and inter-observer reliability was good (*κ* = 0.68), and moderate (*κ* = 0.57), respectively. The callus fracture sign is a useful radiological predictor of progression to non-union and may represent insufficient mechanical stability at the fracture site.

## Introduction

**Fig. 1:**
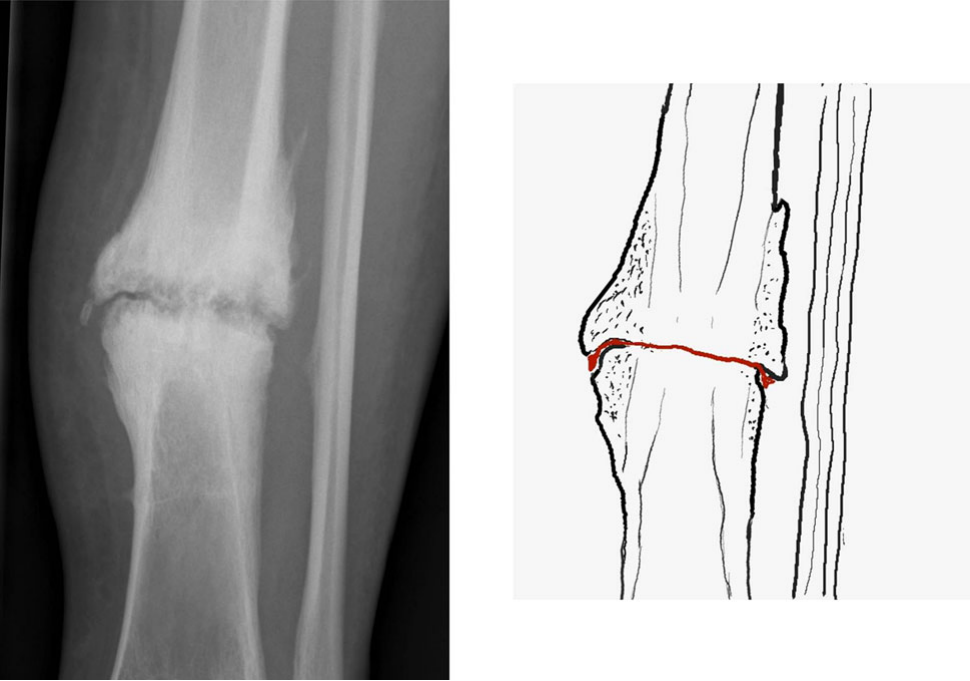
A hypertrophic non-union in a diaphyseal tibial fracture. The line drawing depicts the extension of the fracture line to the periphery of the callus

Fracture union is dependent on the biological environment and the mechanical properties of the fracture site [1]. Hypertrophic non-union can occur in the presence of an appropriate biological response but inadequate mechanical stability. In the context of the Ilizarov method, fractures heal by secondary or indirect bone healing, i.e. in the presence of relative stability provided by the circular fine-wire fixator, the fracture heals by periosteal bony callus (intramembranous ossification) at the periphery of the fracture and fibrocartilaginous bridging callus (endochondral ossification) between bone ends [1, 2]^.^ Here the term ‘bridging callus’ is used to describe the appearance of calcified tissue between the ends of a fracture. Several authors define union as the radiological presence of bridging callus at 3 out of 4 cortices on AP and lateral views [3, 4]. The classic elephant’s foot appearance of a hypertrophic non-union (Fig. 1) results from instability preventing ossification with further cartilaginous material continued to be laid down [1].

**Fig. 2:**
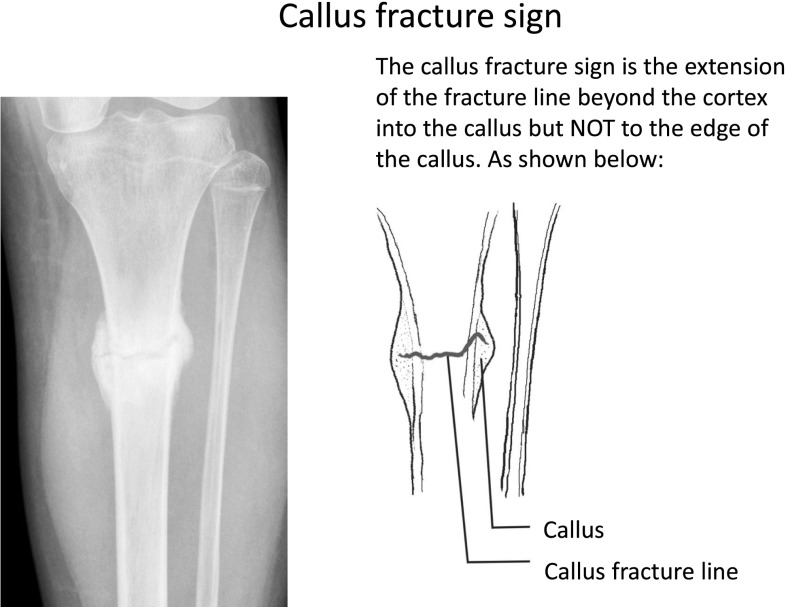
An example of the callus fracture sign. The line drawing highlights the extension of the fracture line beyond the original cortical boundary but not to the periphery of the fracture callus

The incidence of aseptic non-union for fractures of the tibial shaft is 1.5 % in this unit. The senior author has identified a radiological sign in a series of fractures thought to have united but which progressed to established non-union after removal of fixation. In these cases, bridging callus, as defined above, was seen to join the bone ends across the fracture site in more than one view but, on closer examination, the fracture cleavage can be seen to extend beyond the original cortical boundary of the bone but not to the boundary of the bridging callus (Fig. 2). This detail in interpretation of the characteristics of bridging callus has not been identified previously. We have labelled this the ‘callus fracture sign’ and recognise it to be predictive for progression to non-union. The study aims to establish the validity of this radiological sign and its reliability for clinical use.

## Materials and methods

This study was registered with the local audit and research department as a service evaluation, and ethical approval was not required. The study was performed by retrospectively reviewing patients on the Ilizarov database. This database is data prospectively collected from all patients treated by the Ilizarov method.

Between October 2000 and January 2011, a total of 1533 fractures of the tibia treated by the Ilizarov technique were recorded in the database. Fractures of the tibia treated by the Ilizarov technique but went on to an established hypertrophic non-union needing revision frame fixation were included. We excluded patients with confirmed or suspected infection, internal fixation metalwork remaining in situ, atrophic non-unions, and patients with an incomplete set of medical notes or radiographic images. A total of 23 suitable cases were identified; all were closed injuries. A same number of age- and sex-matched patients were identified as controls; these patients had successful union after the same treatment with an Ilizarov frame and had a minimum of 12 months in follow-up. This provided 46 pairs of radiographs.

Radiographs studied were the first images obtained after removal of the Ilizarov all-wire circular fixator. The vast majority of the Ilizarov fixators are removed in an outpatient clinic with the post-removal AP and lateral radiographs taken immediately after. Both AP and lateral views were reviewed in all cases with the 46 pairs of images anonymised and randomised prior to being assessed by six assessors. Three were trauma consultants, two were specialist musculoskeletal radiology consultants, and one was a trauma and limb reconstruction fellow. An example of the ‘Callus Fracture’ sign (Fig. 2) was given with clear written instructions to the assessors for identifying it. An example of an established hypertrophic non-union (Fig. 1) was also provided, and the assessors were permitted to acknowledge whether they felt this was present but it did not count as the ‘Callus Fracture’ sign being present. We have used the term callus fracture sign to describe the extension of the fracture cleavage beyond the limits of the cortex but within the boundary of the callus.

**Fig. 3:**
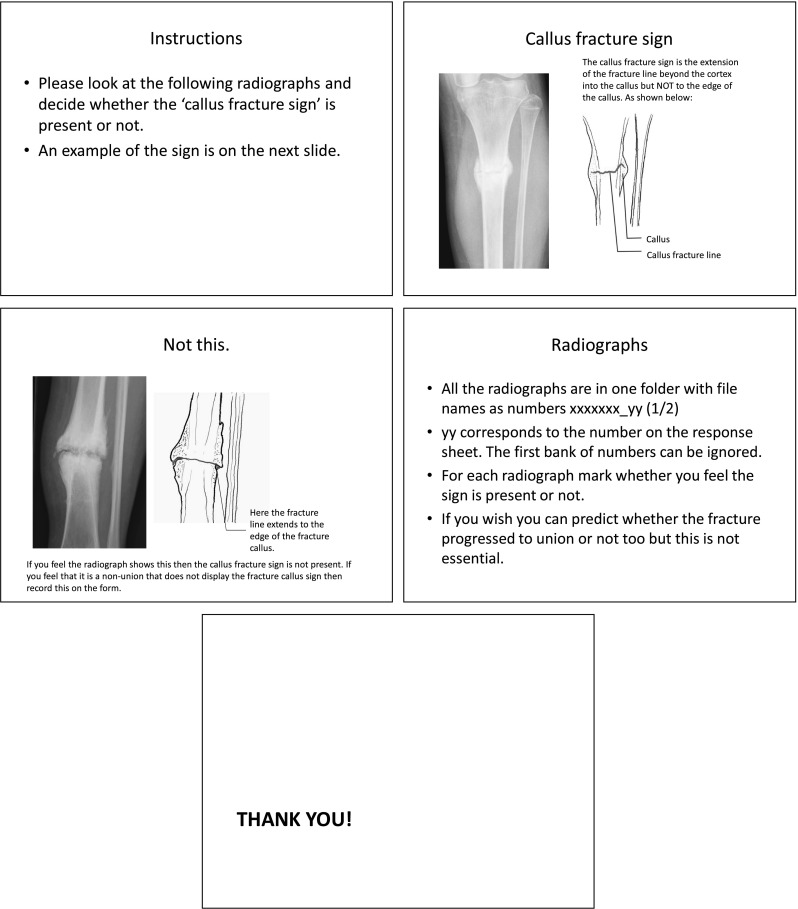
Instructions were provided to the authors as a powerpoint presentation. The slides are shown

Reviewers were asked to identify the presence or absence of a ‘callus fracture sign’ in either view. The instructions given to the reviewers are shown in Fig. 3. Four or more reviewers had to agree on the presence of the sign in order for it to be considered a positive finding. The senior author was asked to review the radiographs on two separate occasions, after a 6-month interval, to allow for analysis of intra-observer reliability.

## Statistics

A contingency table summarising the results was constructed. Pearson’s Chi-square values were calculated using SPSS v17.0 (IBM). True positives (TP) were defined as those with the callus fracture sign who developed a non-union. True negatives (TN) were defined as those without the callus fracture sign who united successfully. False negatives (FN) were those who did not have the callus fracture sign and developed a non-union. False positives (FP) were those who had the sign and united. From this sensitivity (SN), specificity (SP), positive predictive value (PPV), negative predictive value (NPV) and accuracy (AC) were calculated using the following formulas:

$$\begin{aligned} &{\text{SN}} = {\text{TP}}/\left( {{\text{TP}} + {\text{FN}}} \right),\quad {\text{SP}} = {\text{TN}}/\left( {{\text{TN}} + {\text{FP}}} \right), \\ & {\text{PPV}} = {\text{TP}}/\left( {{\text{TP}} + {\text{FP}}} \right),\quad {\text{NPV}} = {\text{TN}}/\left( {{\text{TN}} + {\text{FN}}} \right) \\ & {\text{AC}} = \left( {{\text{TP}} + {\text{TN}}} \right)/\left( {{\text{TP}} + {\text{FP}} + {\text{TN}} + {\text{FN}}} \right) \\ \end{aligned}$$

The *κ* statistic was calculated using Fleiss’ modification for multiple observers using SPSS v17.0 (IBM, USA) [5] A *κ* value of <0.2 was considered poor, 0.21–0.40 fair, 0.41–0.60 moderate, 0.61–0.80 good, 0.81–1.00 very good [6]. Categorical data were analysed using the Chi-square test. A *p* value of <0.05 was considered statistically significant.

## Results

Table 1 shows the results of the senior author alone. Table 2 shows the results when four or more of the assessors considered the callus fracture sign to be present. Presence of the callus fracture sign was agreed in 16 radiographs of the fractures that had progressed to non-union, and 2 of the united fracture group. Using the combined results, sensitivity is 69.6 %, specificity is 91.3 %, positive and negative predictive values are 88.9 and 75.0 %, respectively. Accuracy is 80.6 %. Overall, intra-observer reliability was good (*k* = 0.68) and inter-observer reliability was at the high end of the moderate range (*k* = 0.58).

**Table 1:** Contingency tables summarising results for the senior author

	Senior author review	
	Union	Non-union
Callus fracture sign present	4	14
Callus fracture sign absent	19	9
*χ* ^2^	9.127	
*p* value	0.006

**Table 2:** Contingency table summarising results when four or more of the reviewers independently assessed the callus fracture sign to be present

	Agreement of four or more reviewers	
	Union	Non-union
Callus fracture sign present	2	21
Callus fracture sign absent	16	7
*χ* ^2^	17.889	
*p* value	<0.001	

## Discussion

Perren’s strain theory of fracture healing suggests that the degree of inter-fragmentary strain dictates the type of tissue formed between the fracture ends [1]. It has been demonstrated that the tissue within a non-union site contains mesenchymal progenitor cells that are capable of transforming into cartilage and bone forming cells [7]. When a non-union is deemed to be hypertrophic, increasing stability, for example by fracture distraction, can lead to bony union [8]. Treatment is aimed at increasing mechanical stability and thereby initialising mineralisation of fibrocartilage.

The point at which a tibial fracture is united is a key step in management but is of particular importance in those treated with an Ilizarov frame as it determines when the fixator can be removed. In our unit, like others, this is done when a collection of clinical features are present: bridging callus on the radiographs; the patient is weight-bearing painlessly; and there is no clinically detectable movement at the fracture site. Once deemed a fracture has united, the frame is dynamised then disconnected. If there is no movement between the rings on manual stressing, it is likely no movement has occurred at the fracture site and the frame then removed. These criteria are similar to those described by Sarmiento [9].

These results demonstrate a significant relationship between the callus fracture sign and a requirement for revision surgery. If the callus shows a defect, there may be the tendency for this to break down rather than consolidate if greater stability is not provided. The callus fracture sign is thought to represent a prognostic sign where the visible fracture line on the radiograph evolves into a cleavage plane which would eventually form a hypertrophic non-union when the plane reaches the outer surface of the callus.

Determining fracture union is not straightforward. The original work done on rabbit tibias demonstrated that callus strength peaks when three cortices are bridged by callus [4]. However, in humans radiological union and mechanical strength do not correlate well [10, 11]. As a result, attempts have been made to devise scoring criteria to determine fracture union. Although these scoring criteria have good inter- and intra-observer reliability [12], they correlate poorly with union [10] or have not been validated [13]. Furthermore, these have been designed to assess union in a tibia treated with intramedullary nailing; this is a scenario where the implant is not normally removed after union unlike a circular frame. If these scoring systems are applied to fractures treated with Ilizarov circular frames, there is a risk the fixation may be removed before fracture union is complete then prompting the need for revision surgery. It is unlikely that the callus fracture sign can be applied to fractures treated by internal fixation as, unlike in Ilizarov treatment, it is difficult to subsequently alter the construct to affect the overall rigidity.

CT has been used to diagnose non-union in such cases. The callus fracture sign is similar to CT with respect to sensitivity, specificity and accuracy [14]. Furthermore, one-seventh of the patients in this study underwent unnecessary surgery because of a false-positive CT result. The callus fracture sign has a lower negative predictive value. This may be explained by those with a NPP included cases that had a line across the fracture but not beyond the cortex, and a small number of non-union cases which the assessors felt displayed signs of an established non-union but not the callus fracture sign. If these cases were removed from the analysis, the false-negative rate would be lower and sensitivity, negative predictive value and accuracy would all be improved.

We suggest that those patients demonstrating the callus fracture sign, i.e. the cleavage plane of the fracture extending beyond the original cortical boundary of the bone but remaining within the boundary of the callus as in Fig. 2, should undergo a period of increased fracture stabilization prior to removal of their fixators. In our unit this is done by distraction across the fracture site to place the callus under tension [8].

The limitations of this study include the retrospective design and relatively small number of cases. All the non-unions identified for this study were diaphyseal fractures and the findings cannot be extrapolated to other regions of the tibia. Whilst it may seem logical to extend this clinical sign to metaphyseal and epiphyseal fractures, we do not have the data to confirm this.

The usual progression of treatment with circular fixation is progressive destabilisation of the frame prior to removal. These results suggest the callus fracture line is an indicator that stability may be inadequate and the reversal of this standard protocol to a period of increased stability from the frame, prior to further testing of fracture stability, should reduce the risk of development of hypertrophic non-union.
